# Implementation of the validation testing in MPPG 5.a “Commissioning and QA of treatment planning dose calculations–megavoltage photon and electron beams”

**DOI:** 10.1002/acm2.12015

**Published:** 2016-12-05

**Authors:** Dustin J. Jacqmin, Jeremy S. Bredfeldt, Sean P. Frigo, Jennifer B. Smilowitz

**Affiliations:** ^1^ Department of Radiation Oncology Medical University of South Carolina Charleston SC USA; ^2^ Department of Human Oncology University of Wisconsin–Madison Madison WI USA

**Keywords:** beam modeling, commissioning, dosimetric accuracy, treatment planning system

## Abstract

The AAPM Medical Physics Practice Guideline (MPPG) 5.a provides concise guidance on the commissioning and QA of beam modeling and dose calculation in radiotherapy treatment planning systems. This work discusses the implementation of the validation testing recommended in MPPG 5.a at two institutions. The two institutions worked collaboratively to create a common set of treatment fields and analysis tools to deliver and analyze the validation tests. This included the development of a novel, open‐source software tool to compare scanning water tank measurements to 3D DICOM‐RT Dose distributions. Dose calculation algorithms in both Pinnacle and Eclipse were tested with MPPG 5.a to validate the modeling of Varian TrueBeam linear accelerators. The validation process resulted in more than 200 water tank scans and more than 50 point measurements per institution, each of which was compared to a dose calculation from the institution's treatment planning system (TPS). Overall, the validation testing recommended in MPPG 5.a took approximately 79 person‐hours for a machine with four photon and five electron energies for a single TPS. Of the 79 person‐hours, 26 person‐hours required time on the machine, and the remainder involved preparation and analysis. The basic photon, electron, and heterogeneity correction tests were evaluated with the tolerances in MPPG 5.a, and the tolerances were met for all tests. The MPPG 5.a evaluation criteria were used to assess the small field and IMRT/VMAT validation tests. Both institutions found the use of MPPG 5.a to be a valuable resource during the commissioning process. The validation testing in MPPG 5.a showed the strengths and limitations of the TPS models. In addition, the data collected during the validation testing is useful for routine QA of the TPS, validation of software upgrades, and commissioning of new algorithms.

## 1 Introduction

The recently published Medical Physics Practice Guideline (MPPG) 5.a: “Commissioning and QA of Treatment Planning Dose Calculations–Megavoltage Photon and Electron Beams” provides concise guidance on the commissioning and QA of dose calculations from external beam treatment planning systems.^1^ The guideline outlines a series of validation tests for the dose calculation algorithms used by the treatment planning system (TPS). While the implementation of robust and comprehensive QA programs recommended in other AAPM reports is strongly encouraged, an objective of the MPPG 5.a is to provide the minimum recommendations for dose algorithm commissioning and QA in a clinical setting. The validation testing is divided into four sections: basic photon fields, heterogeneity corrections for photons, IMRT/VMAT, and electron fields. These are sections 5–8, respectively, of the report. With the exception of test 5.1, all validation tests involve computing dose in the planning module of the TPS software and comparing it with measured data. For each test, the associated tolerance value and/or the evaluation criteria are described in the practice guideline. The test suite is more comprehensive than commissioning data in that it comprises volumetric data measurements in addition to profiles and point dose measurements and includes nonstandard field shapes and sizes.

There are a number of previous TPS‐commissioning publications relevant to the present work. In 1993, Van Dyk et al.^2^ offered early guidance on TPS commissioning and QA. In 1998 and 2004, the AAPM and IAEA released Task Group Report No. 53^3^ and TRS 430,^4^ respectively, to provide comprehensive guidelines for acceptance testing, commissioning, and ongoing quality assurance of 3D TPS. Starkschall et al.^5^ described a beam modeling methodology for the convolution/superposition dose calculation algorithm in the Pinnacle TPS, including an assessment of model accuracy using the recommended procedures in TG‐53. In 2001, Venselaar et al.^6^ proposed a set of tests and appropriate tolerances for photon beam dose calculations. In addition, validation tests for nonstandard treatment geometries, inhomogeneous media, MLC modeling, and commissioning have been described.^7^, ^8^, ^9^ The 2008 AAPM Task Group Report No. 106^10^ discusses equipment and procedures to ensure the accurate and self‐consistent collection of commissioning beam data. The 2009 AAPM Task Group Report No. 119^11^ provides guidance and test cases for IMRT commissioning.

A number of previous studies discussed the development of custom tools for automated analysis of commissioning data. Adnani^12^ designed a TG‐106 compliant linear accelerator data management system for physics data acquisition, processing, and validation. Birgani et al.^13^ created a MATLAB program for comparing commissioning measurements and dose distributions from a custom‐made second‐check software. Bergman et al.^14^ used MATLAB to perform an automated 3D gamma analysis comparing treatment planning system and Monte Carlo dose distributions for the HD120 MLC on a Varian TrueBeam linear accelerator. Several authors have reported developing in‐house software for comparing commissioning data to radiochromic film measurements.^15^, ^16^, ^17^ Previous research discussing tools for the analysis of arbitrary validation fields are much less common. Jacqmin et al.^18^ created a comprehensive and efficient system to validate photon dose calculation algorithm accuracy based on the tests recommended in TG‐53. Kim et al.^19^ developed an automated quality assurance procedure for the Pinnacle TPS that assessed beam model accuracy for commissioning beam geometries and additional clinical scenarios. Although all of these tools have contributed greatly to the automation of many commissioning tasks, none were specifically tailored to analyze data captured for MPPG 5.a. We therefore chose to create a new open‐source software tool to share with others who will perform the MPPG 5.a tests. An accompanying spreadsheet is also included to aid the user in analyzing and organizing the results of the MPPG 5.a tests.

The first objective of this work is to report our experience implementing these tests for the Philips Pinnacle (Philips Radiation Oncology Systems, Fitchburg, WI, USA) and Varian Eclipse (Varian Medical Systems, Palo Alto, CA, USA) treatment planning systems on newly commissioned Varian TrueBeam linear accelerators. These two well‐established treatment planning systems have been in use at numerous centers for many years. Two institutions participated in this study: The University of Wisconsin Carbone Cancer Center (UW) and the Medical University of South Carolina Hollings Cancer Center (MUSC). The tests and processes described in this work are applicable to any similar linear accelerator and TPS combination. It is worth noting that the inclusion of two TPSs in this work serves to demonstrate the broad applicability of our methods in implementing MPPG 5.a. Pinnacle and Eclipse were used to model different accelerators at different institutions. Consequently, this work is not intended to compare the two TPSs using the MPPG 5.a validation testing.

The second objective of this work is to present methods and tools that were created to facilitate the delivery and analysis of the validation tests. A set of treatment fields and corresponding multileaf collimator (MLC) patterns, scan queues, and an open‐source MATLAB program were designed. These tools and materials can be disseminated to the physics community to aid with the implementation of the MPPG 5.a guideline.

## 2 Methods

The MPPG 5.a report intentionally allows for flexibility in data acquisition, tools, and processes. The measured data can be acquired with a variety of detectors in solid phantoms, planar or volumetric QA devices, or a scanning water tank. The measurement tools used for this project are summarized in Table 1 alongside each test recommended in the practice guideline. The tests were performed at both institutions and used to validate two different treatment planning systems (UW: Pinnacle v9.8 and MUSC: Eclipse v11.0). UW performed the tests on a TrueBeam STx with high‐definition MLC, while MUSC performed the tests on a TrueBeam with Millennium 120 MLC. A Microsoft Excel spreadsheet was used to track the validation results in a unified format. Time estimates for each step were also recorded in this spreadsheet as part of this project. A template version of the spreadsheet is available for download along with the open‐source software tool discussed in section 2C.

**Table 1:** Summary of MPPG 5.a tests and measurement equipment used in this work

MPPG 5.a section	Test number	Test description	Measurement equipment
5. Photon beams: basic dose algorithm validation	5.1	Physics module versus planning module	None
	5.2	Clinical calibration geometry dose	Scanning water tank; Farmer‐type ionization chambers
	5.3	Planning module dose versus commissioning data	Scanning water tank; scanning ionization chambers
	5.4–5.8	Basic photon beam tests	Scanning water tank; scanning ionization chambers
	5.9	Nonphysical wedge test	MapCHECK2
6. Photon beams: heterogeneity correction validation	6.1	CT‐value‐to‐density calibration	Electron density phantom
	6.2	Heterogeneity correction	Custom phantom; ionization chamber
7. Photon beams: IMRT/VMAT dose validation	7.1	Small field PDD	Scanning water tank; scanning ionization chambers; diode detector
	7.2	Output for small MLC‐defined fields	Scanning water tank; diode detector
	7.3–7.4	TG‐119 and clinical tests	Delta^4^; MapCHECK2
	7.5	External review	Radiochromic film; OSLDs
8. Electron dose validation	8.1–8.2	Basic electron fields and obliquity tests	Scanning water tank; scanning ionization chambers
	8.3	Electron heterogeneity correction	Custom phantom; ionization chamber

### 2.A Preparation

**Figure 1:**
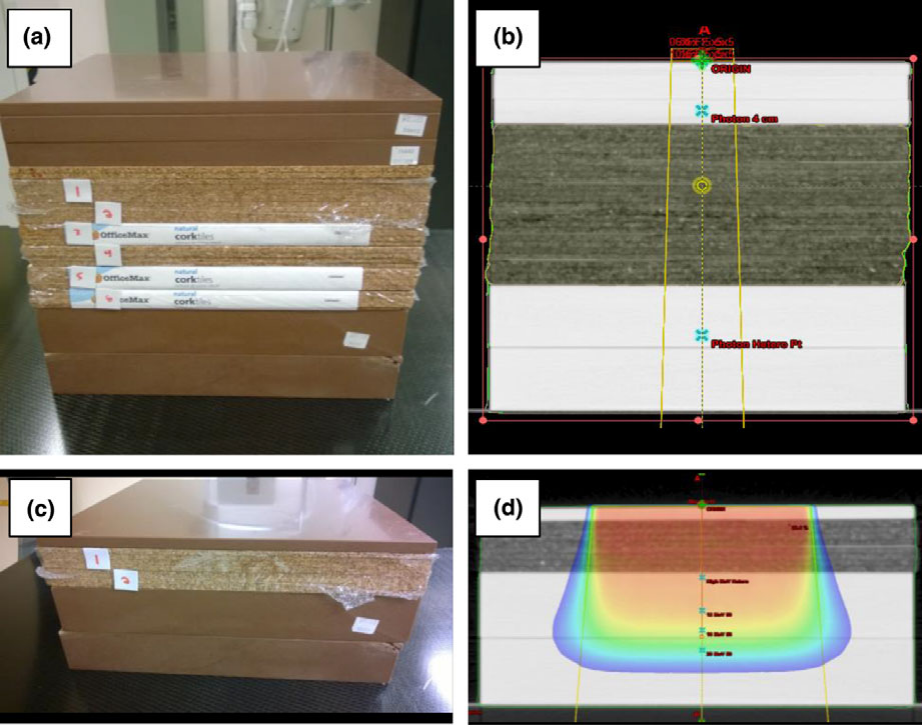
Custom phantoms constructed for MPPG 5.a testing. Custom phantoms were constructed using Solid Water and cork for MPPG 5.a tests 6.2 and 8.3: (a) a phantom with a 13‐cm cork heterogeneity used for test 6.2; (b) CT image of the phantom for test 6.2; (c) a phantom with a 4‐cm cork heterogeneity used for test 8.3 for higher energy electrons; and (d) CT image of the phantom for 8.3 used for higher energy electrons.

Dose calculations were performed at both institutions using the same virtual water tank (a cube of water created in the Eclipse TPS at MUSC). The use of the same virtual water phantom improved the coordination of test planning and data analysis between our centers. For tests 6.2 and 8.3, simple custom phantoms were created from slabs of Gammex RMI Model 457 Solid Water (Gammex RMI, Middleton, WI, USA) and cork as illustrated in Fig. 1. The Delta4 phantom (ScandiDos AB, Uppsala, Sweden) and MapCHECK2 (Sun Nuclear Corporation, Melbourne, FL, USA) were used for the IMRT/VMAT and TG‐119 tests (7.3–7.4). The MapCHECK2 was also used for the nonphysical wedge fields in test 5.9.

The plans created in the TPS for each test were structured to make organization and data export as efficient as possible. In Pinnacle, a separate test patient was created for each of the four primary datasets: water tank, heterogeneous phantom, TG‐119 simulated dataset, and IMRT device. Plans and trials were used to organize the validation tests. In Eclipse, a single test patient was created to store and organize most of the validation tests. Separate courses were created to categorize the tests based on the imaging datasets. A single plan was created in the appropriate course for each water tank test and heterogeneity test, and each plan contained beams with the same aperture but different energies. The plan organization technique has no effect on the dose calculations; it was done for plan and data management purposes only. For both TPSs, dose was calculated using a 2‐mm dose grid and exported separately for each beam. The Pinnacle dose distributions were calculated with the “Adaptive Convolution” algorithm for photons and the “Electron 3D” algorithm for electrons. The Eclipse photon dose was calculated with both “AAA” and “Acuros XB.” The electron dose was calculated with the “eMC” algorithm.

### 2.B Measurements for profile tests

Scanning water tank measurements were used for a number of MPPG 5.a tests, including the basic photon water tank scans (5.4–5.8), small field profile measurements (7.1), and electron profile measurements (8.1–8.2). For each photon test, a scan queue consisting of a percent depth dose (PDD) profile, three inline profiles, and one crossline profile at 10‐cm depth was generated. The inline profiles were measured at *d*_max_, 10 cm, and ≥ 25 cm in accordance with the guideline recommendations. The inline scans were in the direction perpendicular to leaf travel. For electron tests, the scan queues consisted of one PDD, two inline profiles, and two crossline profiles. For each energy, one pair of profiles was measured at the depth of *d*_max_, and the other pair was measured at a depth of *R*_50_. MPPG 5.a does not have specific recommendations for depths at which to measure electron profiles. The depths selected in this work were chosen to assess the beam models at the depth of maximum dose and in the distal falloff region of the electron beam.

**Figure 2:**
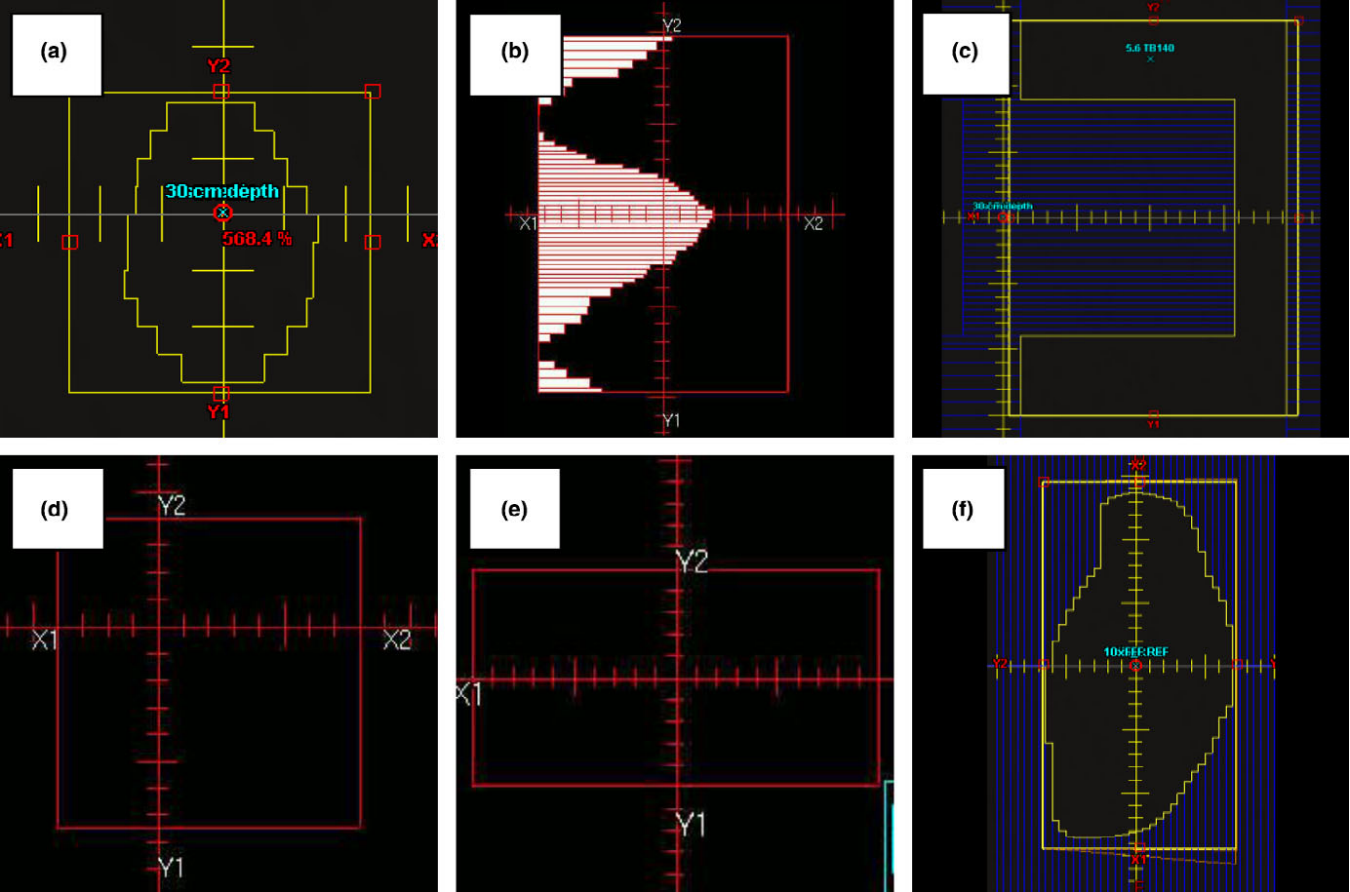
Field apertures for the MPPG 5.a tests in section 5. The field apertures used for tests 5.4–5.9 are shown in the following figures: (a) 5.4 from Eclipse; (b) 5.5 from Pinnacle; (c) 5.6 from Eclipse; (d) 5.7 from Pinnacle; (e) 5.8 from Pinnacle; and (f) 5.9 from Eclipse.

The MLC field shapes for the basic photon tests in section 5 of MPPG 5.a are illustrated in Fig. 2. The small MLC field (test 5.4) was designed to simulate a typical small, nonrectangular MLC‐defined treatment field. The large MLC field (test 5.5) is a larger field with extensive MLC blocking. Test 5.6 is an off‐axis field with the X‐field edge defined by MLCs with maximum allowed leaf over‐travel and the Y‐field edge defined by the jaws. Test 5.7 is an asymmetric field measured at a short source to surface distance (SSD). An SSD of 80 cm was the closest achievable distance at both institutions. Test 5.8 is an MLC‐shaped field at oblique incidence. Depending on the tank placement, the angle of incidence used was either 20 or 30 degrees from the vertical. The scanning measurements were acquired with the IBA Blue Phantom (UW) and the IBA Blue Phantom^2^ (MUSC) beam scanning systems (IBA Dosimetry GmbH, Schwarzenbruck, Germany). The data were collected with IBA CC13 (UW) and IBA CC04 (MUSC) ionization chambers.

**Figure 3:**
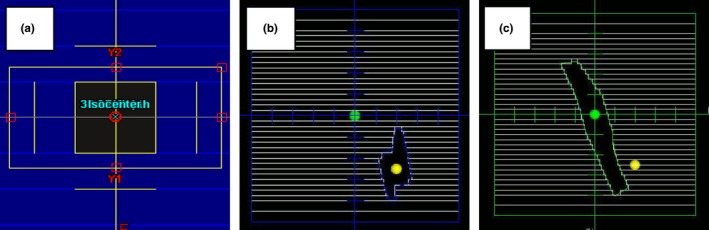
Field apertures for the MPPG 5.a tests in section 7. The field apertures used for tests 7.1 and 7.2 are shown in the following figures: (a) a 1 × 1 cm^2^ MLC field from Eclipse used for 7.1; (b) an irregular, off‐axis MLC‐shaped field from Pinnacle used for 7.2; and (c) a narrow, on‐axis MLC‐shaped field from Pinnacle used for 7.2.

The MLC field shape for test 7.1 is illustrated in Fig. 3. The purpose of test 7.1 is to verify the PDD for an MLC‐shaped field that is 2 × 2 cm^2^ or smaller. Test 7.1 was treated like tests 5.4–5.8: a PDD, three inline profiles, and one crossline were measured. The data were collected with the IBA EFD diode (UW) and the Sun Nuclear Edge diode (MUSC).

**Figure 4:**
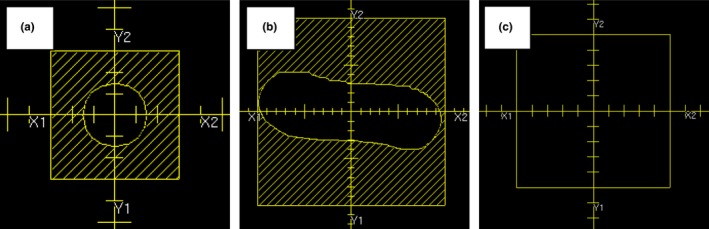
Field apertures for the MPPG 5.a tests in section 8. The field apertures used for tests 8.1 and 8.2 are shown in the following figures: (a) a 3‐cm diameter circular cutout in a 6 × 6 applicator from Pinnacle used for 8.1; (b) a long, narrow cutout in a 20 × 20 applicator from Pinnacle used for 8.1; and (c) a standard 10 × 10 electron field from Pinnacle used for 8.2.

The electron beam validation tests used a combination of custom cutouts and standard open applicators. The Cerrobend cutout shapes for test 8.1 are shown in Fig. 4. One cutout was a 3‐cm‐diameter circle in a 6 × 6 applicator, and the other was a long, narrow curved shape in a 20 × 20 applicator. The cutouts represent two clinically relevant shapes for which the dose calculation is more difficult due to the narrow field dimensions. The applicators for these cutouts span the range of sizes most often used in our clinics. All of the electron beams were scanned with the gantry and collimator at the 0° IEC position. We measured a PDD, two crossline profiles, and two inline profiles at both 100‐ and 105‐cm SSD for each beam energy. Test 8.2 is a measurement of a standard cone at oblique incidence. Both institutions used their reference 10 × 10 cm^2^ cone for this test. Depending on the tank placement, the angle of incidence was either 20° or 30° from the vertical. The measurements included a PDD, two crossline profiles, two inline profiles, and one profile along the central axis of the oblique beam. The scanning measurements for all of these tests were taken with CC13 (UW) and CC04 (MUSC) ionization chambers.

The scanning tank measurements from tests 5.4–5.8, 7.1, and 8.1–8.2 were compared to treatment planning system calculations using a custom MATLAB program called the Profile Comparison Tool (PCT). The PCT is discussed in more detail in the next section. A relative 1D gamma analysis was performed with a gamma criterion consistent with the tolerance values in MPPG 5.a. All of the gamma analysis in this work uses a global dose difference relative to the maximum measured dose. The photon PDDs were normalized at a 10‐cm depth while the electron PDDs were normalized at the depth of maximum dose. The inline and crossline profiles were normalized on the central axis whenever possible. For tests 5.5 and 5.6, the central axis is blocked so the normalization point was moved to a central location in the open field.

### 2.C The profile comparison tool

A MATLAB program called the Profile Comparison Tool (PCT) was created to compare measured and calculated dose profile data. The PCT accepts profile data from scanning water tank systems and 3D DICOM‐RT Dose files from commercial TPS. The PCT can efficiently compare multiple beam profiles (depth dose, inline, crossline, and diagonal) with a single 3D DICOM‐RT Dose file. The first step in this process is to register the coordinate systems of the scanning tank and the 3D DICOM‐RT file. This is done by either placing a TPS reference point at the origin of the scanning tank or by manually entering the offset between the TPS and the scanning tank origins. The calculated dose at each location in the profile is determined from the 3D DICOM‐RT Dose file using a 3D cubic interpolation algorithm. Finally, the two profiles (measured and calculated) are compared using a 1D gamma analysis according to the technique described by Low et al.^20^ The user may specify a number of analysis settings, including gamma analysis options and normalization methods.

**Figure 5:**
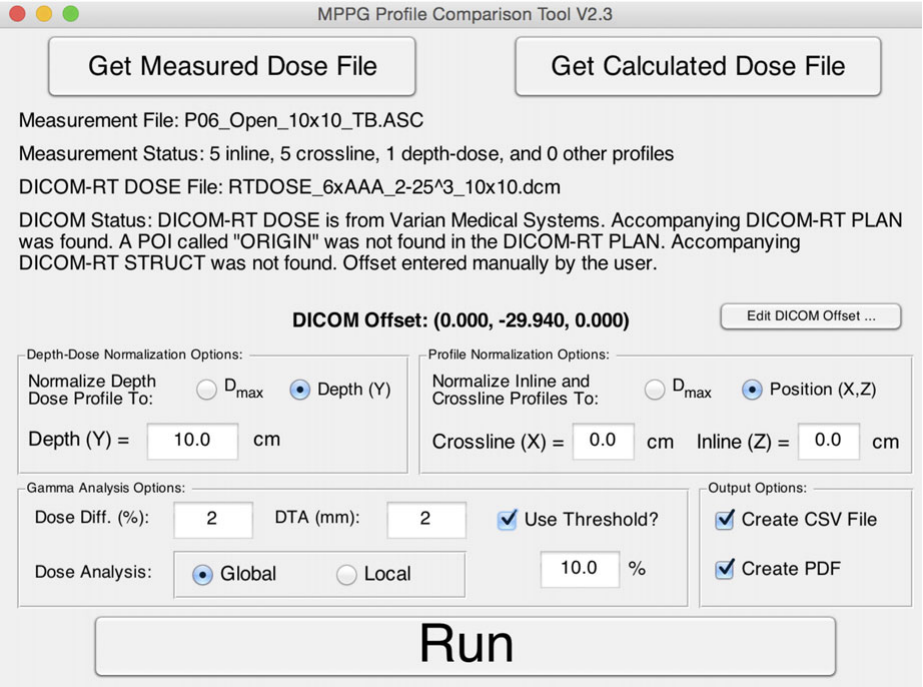
The graphical user interface for the Profile Comparison Tool is shown. The interface allows the user to load measured and calculated data, adjust gamma analysis settings, run a gamma analysis on the imported data, and specify the output format.

**Figure 6:**
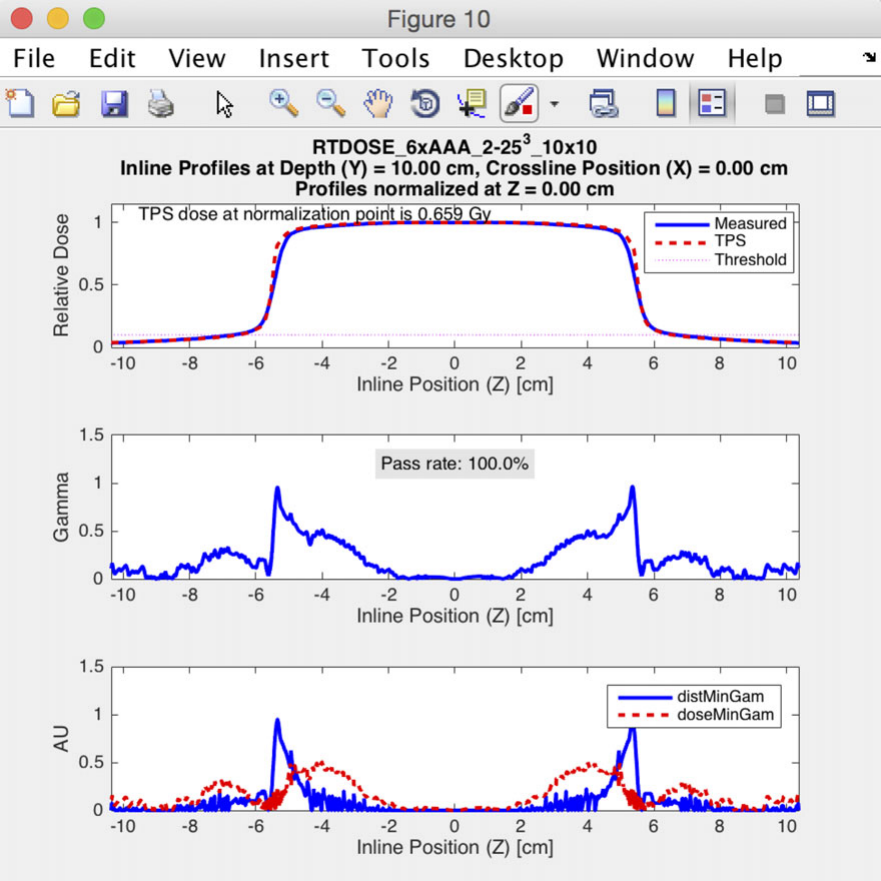
An example of the output from the Profile Comparison Tool is shown. The analysis was performed for a 10 × 10 cm^2^ field for a 6 MV photon beam using a 2% global/2 mm gamma analysis criterion. The figure shows the results for an inline profile measured at 10‐cm depth.

The program has an easy‐to‐use graphical user interface (Fig. 5). A sample output from the PCT is shown in Fig. 6 and includes overlaid dose profiles, gamma as a function of position, distance error at minimum gamma, and dose error at minimum gamma. The last two parameters (distance error and dose error at minimum gamma) give the user an indication if the resulting gamma value is dominated by a position error or a dose error. The results can be exported into a tabular CSV file and a PDF file showing the plots of the profile comparisons. The PCT (MATLAB source code and an executable version of the code), a DICOM file renaming tool, and a set of user instructions are available for download at https://github.com/open-source-medical-devices/mppg. The organizational and analysis spreadsheet is also available for download at this site.

### 2.D Measurements and analysis for nonprofile tests

The point dose measurement tests (5.2, 6.2, 7.2 and 8.3) are relatively straightforward, and the measurement data were easily analyzed using our spreadsheet. Test 5.2 is an important test to verify the absolute dose specification in the planning system. A beam equivalent to the calibration geometry (e.g., 10 × 10 cm^2^ at 100 cm SSD) was created in the TPS to ensure that the dose per MU matches the measured value at the calibration depth (e.g., 10 cm). For the heterogeneity correction tests for photons and electrons (6.2 and 8.3), relative dose was measured above and below the heterogeneity. For the photon tests, measurements were taken at least 4 cm downstream of interfaces to ensure transient charged particle equilibrium. For the electrons, the amount of Solid Water and cork was changed for each energy so that ion chamber readings could be taken near the low‐gradient peak of the depth‐dose distribution. For the small MLC‐defined field output check (test 7.2), either a Sun Nuclear Edge detector or an IBA EFD was used to measure the output factor in a water phantom. The field shapes are shown in Fig. 3. The fields were designed to be smaller in one dimension than the smallest field size for which an output factor is defined in the TPS. This allowed us to explore the limitations of our models for small, irregular field shapes common in IMRT and VMAT.

For the verification of enhanced dynamic wedges (EDW) (test 5.9) a large MLC‐shaped field, as shown in Fig. 2f, was measured with the MapCHECK2 2D diode array. Measurements were taken at 5‐cm, 10‐cm, and 20‐cm depth for the 60‐degree wedge. These measurements were taken twice: once with Y1‐jaw motion and once with Y2‐jaw motion. In addition, all other wedge angles were measured at a 10‐cm depth with the Y1‐jaw motion EDW. The HU‐value‐to‐density calibration test (6.1) was checked by scanning the Gammex RMI 467 Tissue Characterization Phantom and confirming the CT values are reported correctly in the TPS.

The TG‐119 cases and clinical IMRT/VMAT cases (tests 7.3 and 7.4) were measured with commercial IMRT QA devices. The measurements were analyzed with the commercial software specific to the QA phantoms. Gamma evaluation criteria of 2%/2 mm and 3%/3 mm were used to evaluate the models per the MPPG 5.a recommendation. A dose comparison was considered passing at a given gamma criterion if the gamma passing rate was greater than 95%. Finally, an external review is recommended as a validation test for IMRT/VMAT delivery. MUSC and UW verified the accuracy of their treatment planning systems using the Imaging and Radiation Oncology Core (IROC) Spine Phantom and Liver Phantom tests, respectively. These tests were planned, delivered, and analyzed according to IROC specifications.

### 2.E Implementation of tolerance values and evaluation criteria

Section 2.CB of MPPG 5.a discusses the distinction between tolerance values and evaluation criteria. The guideline prescribes tolerance values when there are widely accepted values available in the literature (such as for basic photons measurements under conditions of charged particle equilibrium, etc.). The guideline recommends evaluation criteria for IMRT/VMAT model assessment because widely accepted tolerance values are not available. As such, the results in this manuscript are divided along these lines. Tolerance values in the MPPG 5.a. Tables 5 (basic photons), 6 (photon heterogeneity), and 9 (electrons) are used in this work. For IMRT/VMAT evaluation, we used the evaluation criteria listed in MPPG 5.a. (Table 8).

## 3 Results

The validation tests outlined in MPPG 5.a were performed at two institutions with newly created tools. The validation experience resulted in more than 200 water tank scans and more than 50 point measurements per institution. Time estimates for preparation, measurement, and analysis activities are summarized in Table 2. Time estimates for each test are summarized in Table 3. The time estimates shown are for a linear accelerator with four photon energies, five electron energies, and no physical wedges. Overall, we estimated the testing took approximately 79 person‐hours, of which 26 person‐hours required time on the machine. The rest of the time was dedicated to preparation and analysis, which, in general, can be completed simultaneously by multiple physicists. The preparation, measurement, and analysis for tests 7.3, 7.4, and 7.5 required roughly half of the total test time. These tests involved validating the dose calculation algorithm for IMRT and VMAT using TG‐119 plans, end‐to‐end testing, and an external review. These time estimates were based on the following: performing TG‐119 measurements for four plans per photon energy using planar dosimetry, performing end‐to‐end testing on one clinical plan per energy, and irradiation of the IROC spinal SRS phantom for a single energy.

**Table 2:** Time estimates for MPPG 5.a validation testing by activity

Activity	Description	Time (person‐hr)
Preparation	Create plan in TPS	17.2
	Create scan queues	1.2
	Create spreadsheet	4.3
	CT imaging of phantoms	2.0
	Subtotal	24.7
Measurement	Ion chamber measurements in phantom	5.0
	QA device measurements	11.5
	Scanning measurements	8.5
	Miscellaneous measurements	1.0
	Subtotal	26.0
Analysis	Analysis with profile comparison tool	11.6
	Analysis with QA software	5.5
	Data processing in scanning tank software	4.5
	Miscellaneous data analysis	6.5
	Subtotal	28.1
Total	Total	78.8

**Table 3:** Time estimates for MPPG 5.a validation testing by test

Test ID	Time (person‐hr)
5.1	0.0
5.2	0.3
5.3	8.5
5.4	2.7
5.5	2.4
5.6	2.4
5.7	2.4
5.8	2.4
5.9	1.6
6.1	1.0
6.2	3.7
7.1	1.2
7.2	1.2
7.3	16.0
7.4	7.0
7.5	15.0
8.1	4.2
8.2	2.5
8.3	4.4
Total	78.8

### 3.A MPPG 5.a tests with tolerance values

**Figure 7:**
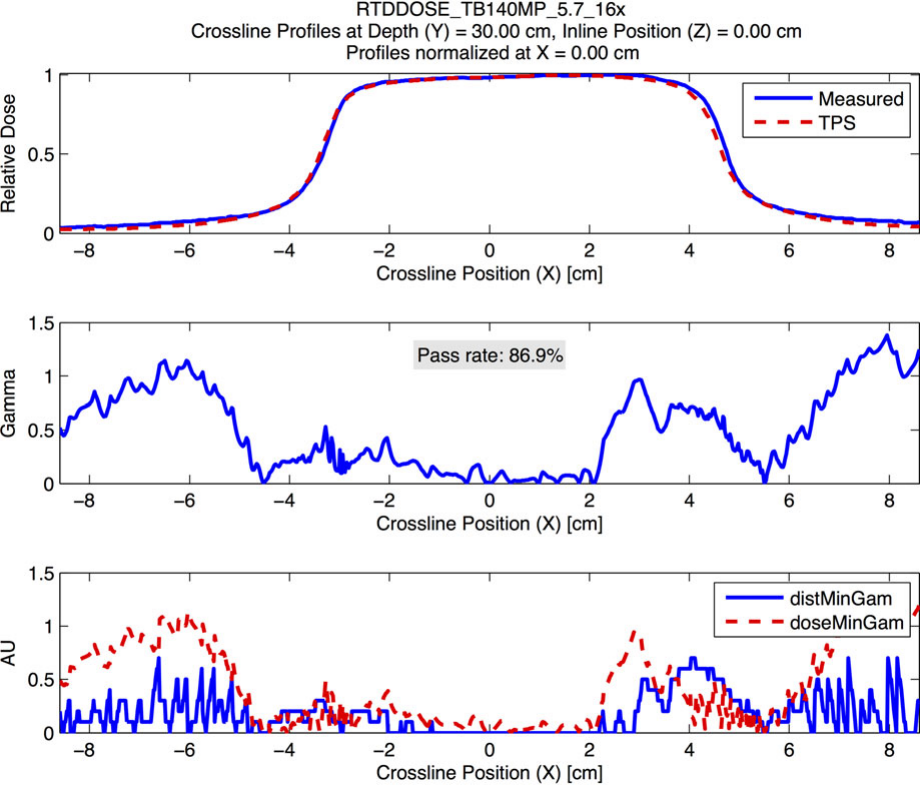
An example demonstrating the modeling errors from Eclipse Acuros XB for the out‐of‐field dose at deeper depths. The figure shows a crossline profile at 30‐cm depth for a 16 MV photon beam for test 5.7. The analysis was performed using a 2% global/2 mm gamma analysis criterion. The figure demonstrates systematic underestimation of the out‐of‐field dose by Eclipse Acruos XB at deeper depths.

The results of all the validation tests in MPPG 5.a sections 5 and 8 meet the tolerances for the basic photon and electron models for both the Pinnacle and Eclipse TPS. Similarly, the results from the heterogeneity correction tests in sections 6 and 8 also meet the tolerances suggested in MPPG 5.a. One of the benefits of using the PCT for analyzing the basic photon and electron tests is that it allowed us to discover characteristics of our models that are not explicitly evaluated using the MPPG 5.a tolerances. For example, there were 25 profiles in tests 5.4–5.9 whose dose comparisons for Eclipse had gamma passing rates lower than 95% at 2%/2 mm. The poor agreement in these profiles is largely confined to the low‐dose tails of the profiles, as shown in Fig. 7. Acuros XB accounted for 19 of the poor results while AAA accounted for the remaining 6. MPPG 5.a recommends a tolerance of 3%/3 mm when evaluating low‐dose and high‐gradient regions of a dose distribution for basic photon tests. When this tolerance is applied, all of the profiles have gamma passing rates above 95%. The use of the recommended tolerances in MPPG 5.a makes the out‐of‐field dose discrepancy in Eclipse more difficult to detect.

**Figure 8:**
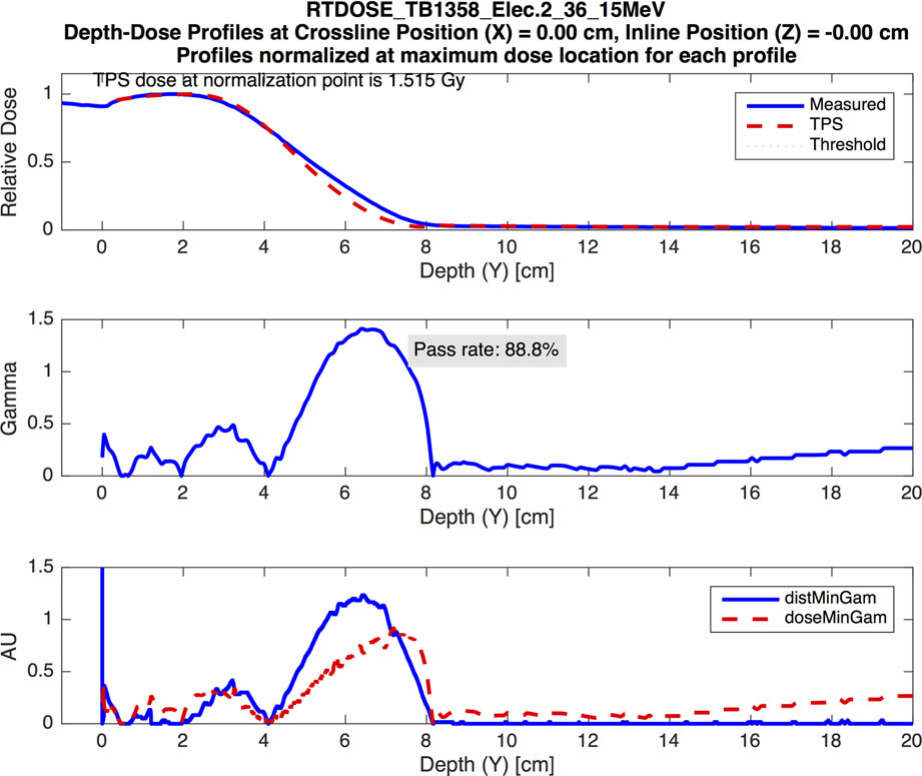
An example demonstrating the small electron field PDD modeling errors from Pinnacle. The figure shows a PDD curve for a 15‐MeV electron beam delivered through the 3‐cm‐diameter cutout. The analysis was performed using a 3% global/3 mm gamma analysis criterion. The figure demonstrates poor modeling beyond the depth of *R*_50_ by the Pinnacle model.

**Figure 9:**
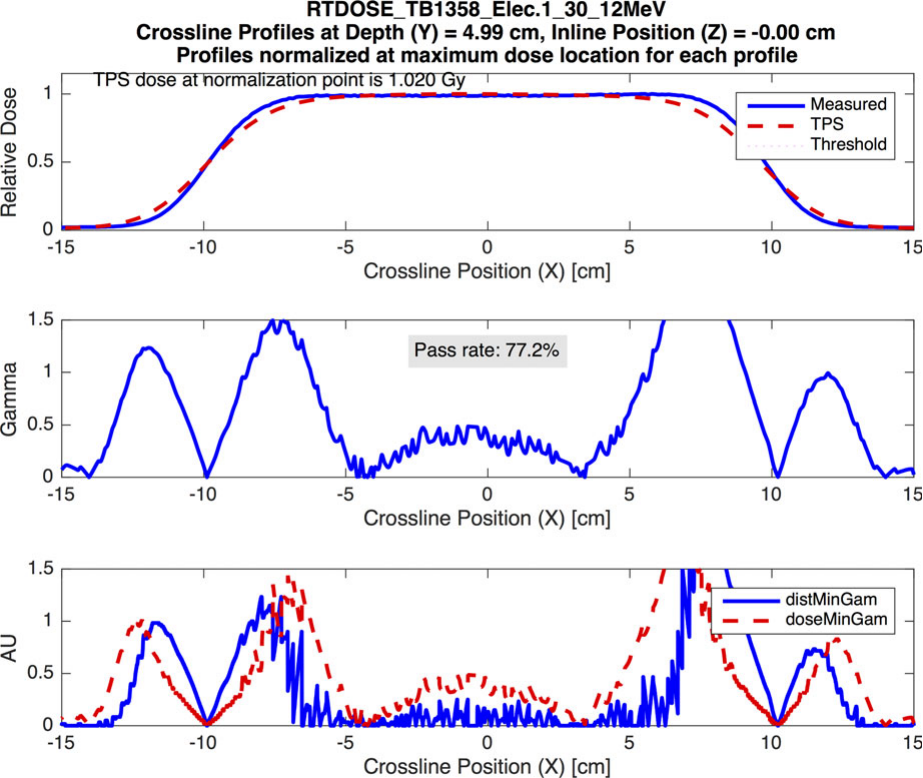
An example demonstrating the modeling errors from Pinnacle for the large electron field profiles measured at the depth of *R*_50_. The figure shows a crossline profile at the depth of *R*_50_ (4.99 cm) for a 12‐MeV electron beam delivered through the large cutout shape. The analysis was performed using a 3% global/3 mm gamma analysis criterion. The figure demonstrates poor modeling in the shoulders and tails of the profile by the Pinnacle model.

The PCT also allowed us to discover dose discrepancies in our electron models that were not uncovered using the MPPG 5.a tolerances. For example, two of the measured PDDs for the small cutout in test 8.1 showed dose discrepancies for Pinnacle beyond *R*_50_, which resulted in gamma passing rates below 95%. An example is shown in Fig. 8. The other PDDs for the small cutout exhibited a similar discrepancy, but the magnitude was small enough that the measurements met the 3%/3 mm gamma criterion. All of the PDDs for the large cutout in test 8.1 and the open cutout in 8.2 had gamma passing rates at or near 100%. This suggests that Pinnacle may not model PDDs accurately beyond the depth of *R*_50_ in the absence of lateral electron equilibrium for small fields like the small cutout used in 8.1. The MPPG 5.a recommendations state that the tolerances should only be applied to high‐dose/low‐gradient regions of the dose distribution, so this dosimetric disagreement does not violate any tolerances in the guideline. Nonetheless, we found this to be valuable information about our model. Similarly, 11 profiles measured for the large cutout in test 8.1 and 11 profiles measured for test 8.2 had gamma passing rates lower than 95%. All of these profiles were measured at the depth of *R*_50_, which is in the high‐gradient tail of the dose distribution. The discrepancy was largest in the shoulders and tails of the profiles, as demonstrated in the example shown in Fig. 9. These profiles are also outside of the high‐dose/low‐gradient region of the dose distribution, so this dosimetric disagreement does not fail any recommended tolerances in the MPPG 5.a.

### 3.B MPPG 5.a tests with evaluation criteria

MPPG 5.a recommends relatively tight evaluation criteria for the validation tests in section 7 since (1) no widely acceptable criteria are published for small fields or IMRT/VMAT, and (2) an objective of the validation tests is to reveal weaknesses in the models. We evaluated the tests in section 7 using gamma criteria of 2%/2 mm and 3%/3 mm. Table 4 summarizes the results of these tests for both criteria. Test 7.1 evaluated PDDs for very small fields. The measured PDDs matched the models for Pinnacle and Eclipse within 2% beyond the buildup region for all beams. Our implementation of test 7.2 used two small fields (Figs 3b and 3c), neither of which provide charged particle equilibrium near the peak dose. Consequently, the measurement locations were in high‐dose/high‐gradient regions. There was no corresponding tolerance or evaluation criteria listed in the guideline for this situation (Table 8 of MPPG 5.a lists a tolerance of 2% for high‐dose/low‐gradient measurements). Nine of the 12 dose comparisons for test 7.2 were within 2% and 11 of 12 were within 3%.

**Table 4:** Summary of MPPG 5.a validation results for tests 7.1–7.4

Test ID	Total measurements	Passing measurements		Passing rate (%)	
		2%/2 mm	3%/3 mm	2%/2 mm	3%/3 mm
7.1	44	44	44	100	100
7.2	12	9	11	75	91.7
7.3	40	40	40	100	100
7.4	57	48	54	84.2	94.7

At MUSC, all of the TG‐119 test cases achieved a gamma passing rate greater than 95% at both 2%/2 mm and 3%/3 mm. In lieu of TG‐119 test cases, UW ran an extensive set of clinical test cases in test 7.4. UW measured 10 clinical IMRT and VMAT cases containing 35 fields and 10 composite dose distributions. Overall, seven fields and one composite dose distribution failed at 2%/2 mm. Two individual fields and one composite dose distribution failed at 3%/3 mm. MUSC measured 12 clinical IMRT and VMAT plans containing 48 fields. Only composite dose distributions were analyzed. Overall, one of the 12 plans failed at 2%/2 mm and zero of the 12 plans failed at 3%/3 mm. The clinical cases were not the same at the two institutions. The end‐to‐end external review tests (test 7.5) passed for both TPSs.

## 4 Discussion

MPPG 5.a provides a simple, flexible framework for commissioning and validation of TPS dose calculation algorithms. While validating a TPS for clinical use can be an arduous task, we found our implementation of MPPG 5.a to be a valuable exercise. The TPS models for Pinnacle and Eclipse met the minimum tolerances in MPPG 5.a, and this makes the models clinically acceptable per the guideline. By performing the recommended validation tests, we learned about the strengths and weaknesses of our models, which are summarized in this section. We also share insights from our experience in the hopes that future adopters of the MPPG 5.a can benefit from our tools and test implementation. Moreover, since all MPPG are intended as “living documents” with 5‐year sunsets, we hope this feedback will prove valuable in future versions of the TPS commissioning guideline.

Tests 5.1–5.3 serve as sanity checks and do not involve additional measurements beyond those acquired at commissioning. The beam calibration geometry is reproduced in the TPS in test 5.2 to validate the absolute dose specification. This simple test is particularly important because it can prevent a systematic error in absolute dose specification. For the static photon validation tests (5.4–5.8), the PCT was exceptionally valuable because it could be run quickly using multiple gamma criteria. The tests at both institutions found gamma passing rates exceeding 95% at 2%/2 mm for the PDDs and the high‐dose region of the profiles. However, the validation testing revealed limitations with the out‐of‐field dose modeling for Eclipse, which required us to reanalyze the profiles at 3%/3 mm to verify that the out‐of‐field dose agreement met the tolerance specified in MPPG 5.a. For Eclipse, the out‐of‐field dose modeling was poor for both the AAA and the Acuros XB algorithm, but the results were worse for Acuros XB. The out‐of‐field dose modeling showed the greatest discrepancy between measurement and calculation at deeper depths. Underestimation of out‐of‐field dose has been documented in the literature for both Eclipse AAA^21^, ^22^ and Pinnacle.^23^ These studies report local dose differences in the out‐of‐field region as large as 50%. To our knowledge, our study is the first to indicate the underestimation of out‐of‐field dose may be worse for Eclipse Acuros XB than AAA. Accurate modeling of the out‐of‐field dose can be critical when calculating dose for certain treatment situations, including fetal dose or implantable cardiac devices.

Test 6.1 verified that the HU‐value‐to‐density calibration was appropriately applied for both TPS. Test 6.2, which validates heterogeneity corrections for photon beams, was a simple and useful test. The Eclipse AAA and Pinnacle CS algorithms were accurate to within 2% for all energies both above and below the heterogeneity. Due to the unique characteristics of Acuros XB, results of test 6.2 depend upon whether the phantom materials were overridden to non‐biological materials in the TPS. The Eclipse Acuros XB algorithm assigns biological materials to each voxel in the CT dataset.^24^ When the dose calculation was performed on the phantom using the dose‐to‐medium reporting method and no material overrides, the dose differences exceeded 2%. For this dose calculation method, the dose at the ion chamber locations was calculated in a combination of “skeletal muscle” and “cartilage” materials that are automatically assigned by Acuros XB. The maximum difference between Acuros XB and the measured dose was 2.2% above the heterogeneity and 2.6% below the heterogeneity. The results improved significantly when the Solid Water and cork regions in the TPS were overridden to the “water” and “cork” materials, respectively. The maximum difference between Acuros XB and the measured dose was 1.3% above the heterogeneity and 0.6% below the heterogeneity when material overrides were used in the dose calculation. We learned that water‐equivalent phantoms should be manually overridden to the “water” material for accurate dose calculation when using Acuros XB.

Pinnacle and Eclipse performed well on the small static field validation tests 7.1 and 7.2. The PDDs and profiles in test 7.1 had gamma passing rates of 95% and higher at 2%/2 mm. UW used these tests to fine‐tune the Gaussian height and Gaussian width model parameters in Pinnacle. MUSC used this test to verify that the “Source Size” parameters in Eclipse were appropriately set. The point dose measurements in test 7.2 were within 3% of the calculations with only one exception. In hindsight, our test shapes may have been more complicated than what is recommended in MPPG 5.a. Our intention was to use shapes that were reminiscent of MLC segments in IMRT and VMAT plans. In practice, it is very difficult to measure output factors for these odd shapes due to the lack of charged particle equilibrium and the sensitivity of the measurement to detector positioning.

Tests 7.3–7.5 accounted for most of the testing time because the plans had to be optimized after the static field model was ready. In addition, the model parameters for IMRT and VMAT typically needed to be iteratively adjusted, resulting in a more involved analysis process. Both institutions found that they could achieve excellent profile agreement for static fields, but that this was not sufficient for optimizing dosimetric agreement for IMRT and VMAT. Tests 7.3 and 7.4 were used to refine the MLC model parameters, including MLC transmission for both TPS; the dosimetric leaf‐gap for Eclipse; and leaf tip radius, tongue‐and‐groove width, interleaf leakage transmission, and the rounded leaf offset table for Pinnacle.

Once these parameters were optimized, both institutions used clinical plans designed for test 7.4 to verify their beam models for IMRT and VMAT. We discovered that our models could not obtain the same level of agreement across the full range of field sizes used in clinical practice. In particular, we initially found that our models did not perform as well for IMRT and VMAT plans that treated large volumes, requiring further adjustment of the MLC model parameters. One limitation of the TG‐119 plans is that the volumes are quite small and do not require some of the techniques used clinically to treat large volumes. It is important to use test 7.4 to verify cases that represent the extremes seen in clinical practice. As discussed in the Results, one of the clinical test cases failed to pass at the 3%/3 mm gamma criterion. This plan was overmodulated and represented a case that would need to be replanned in clinical practice. Both institutions successfully completed test 7.5 using anthropomorphic phantoms obtained from IROC. The independent third party verification process provided additional confidence in the TPS models before releasing the new treatment delivery systems for clinical use.

MPPG 5.a recommends tolerances for the electron validation tests (8.1 and 8.2), but states that the tolerances should only be applied in high‐dose/low‐gradient locations where lateral electron equilibrium is present. The profile comparisons for Eclipse eMC had high gamma passing rates for all PDDs and profiles, even in high‐gradient and low‐dose regions. The Pinnacle Electron 3D algorithm performed well for all of the measurements made in high‐dose/low‐gradient regions, but we found areas with larger dose differences outside of these regions. For example, the tails and shoulders of the profile measurements at a depth of *R*_50_ (high‐gradient distal falloff region) had noticeably worse agreement in Pinnacle compared to Eclipse eMC. In addition, the electron PDDs for the small electron cutout exhibited poor agreement beyond *R*_50_, a region characterized by a high gradient and a lack of lateral electron equilibrium. The electron heterogeneity validation test (8.3) resulted in good agreement between our dose calculation algorithms and the measurements for both TPS, easily meeting the 7% recommended tolerance.

In addition to the overall validation of our clinical systems, the second objective of this project was to present tools and methods used by our institutions. The development of the PCT required a lot of work up front but proved to be an invaluable tool that efficiently analyzed a large volume of profile data using flexible evaluation criteria. It is our hope that by sharing this tool (and the accompanying spreadsheet), others can add to its utility and ease of use. Test design is another important aspect of MPPG 5.a. The guideline does not explicitly define test fields, but rather defines a limited scope of validation to be done. Initially, we considered tests that were more rigorous and beyond the scope of MPPG 5.a (e.g., measuring at additional electron depths and creating very small test shapes for 7.2). As expected, these more challenging geometries highlighted weaknesses of the algorithms and the limits of our measurement abilities in very small fields. Ultimately, all of the validation tests showed that our algorithms met the widely accepted, published tolerances quoted in sections 5, 6, and 8 of MPPG 5.a. The use of a 2%/2 mm gamma criteria and more rigorous small field testing was a good complement to the limited dataset recommended by vendors for commissioning. For users who wish to scrutinize their TPS models further, we recommend comparing your results to previous work on the topic of validation found in the literature.^2^, ^3^, ^4^, ^5^, ^6^, ^7^, ^8^, ^9^, ^10^, ^11^ Finally, we wanted to note what appears to be an error in MPPG 5.a to future users of the guideline. Table 8 in MPPG 5.a uses the term “tolerance” when it is clear from the text of the guideline (particularly section 2.CB) that the values are meant to be evaluation criteria. We suggest this column heading be changed to “evaluation criteria” to more accurately reflect the intent of the more rigorous IMRT/VMAT gamma criteria.

We recommend a slightly different organization of the tests in MPPG 5.a in future updates to the guideline. It is more fitting that sections 5 and 8 be grouped together. These tests cover basic photon and electron dose calculation in simple water geometries and are likely to be measured using similar tools. Within this section, we recommend the creation of a test like 5.2 for electrons to verify the absolute dose calculation at the reference point. We further recommend that section 6 and test 8.3 be included in one section dedicated to heterogeneity corrections for photons and electrons. These tests will employ similar phantoms and measurement devices for most users. Finally, section 7, which is dedicated to small fields and IMRT/VMAT, should remain intact as the third major section of validation tests.

The MPPG 5.a testing is done at the time of commissioning. It forms an integral part of the beam model validation process and routine QA. Both UW and MUSC have used their MPPG 5.a datasets during TPS upgrades after the original testing. Once the initial measurements are obtained, the established tools and methods for data export and analysis allow the dose calculation algorithm to be recalculated and reevaluated quickly. In addition, both UW and MUSC have used their MPPG 5.a datasets to commission Mobius3D, a software package that uses a 3D collapsed‐cone convolution‐superposition algorithm to perform secondary plan checks (Mobius Medical Systems, Houston, TX, USA). The use of the MPPG 5.a tests allowed us to directly compare the accuracy of Mobius3D to our treatment planning systems.

## 5 Conclusion

Implementing the validation tests in MPPG 5.a was a valuable exercise. While it represents a significant time commitment, the resulting infrastructure has been useful for subsequent software and hardware validation tests, as well as routine QA at our institutions. We have presented tools and processes to efficiently perform the MPPG 5.a tests and organize the results. Most of the tools and processes that we used are applicable, or easily adaptable, to other radiation oncology clinics. In particular, the Profile Comparison Tool was designed to be as flexible as possible and work with a number of treatment planning systems and scanning water tank configurations. It is our hope that with only minimal adaptation, the amount of time needed to implement MPPG 5.a testing will be significantly decreased by using the tools we have shared. Overall, MPPG 5.a made QA of TPS easier, more robust, and more uniform across the clinic.

The MPPG 5.a tests serve as an opportunity for physicists to interrogate their beam models in a wide variety of geometries and learn if there are particular geometries in which their beam models perform poorly. The discovery of poorly modeled geometries provides useful information for physicists, who can advise against certain plan designs or perform patient‐specific measurements for certain delivery parameter combinations.
